# Relationship between animal health and livestock farmers’ wellbeing in Ghana: beyond zoonoses

**DOI:** 10.1186/s12889-023-16287-2

**Published:** 2023-07-14

**Authors:** Francis Sena Nuvey, Daniel T. Haydon, Jan Hattendorf, Kennedy Kwasi Addo, Gloria Ivy Mensah, Günther Fink, Jakob Zinsstag, Bassirou Bonfoh

**Affiliations:** 1 Swiss Tropical and Public Health Institute, Kreuzstrasse 2, Allschwil, 4123 Switzerland; 2 Faculty of Medicine, University of Basel, Klingelbergstrasse 61, Basel, 4056 Switzerland; 3 Centre Suisse de Recherches Scientifiques en Côte d’Ivoire, Abidjan, BP 1303 Côte d’Ivoire; 4 School of Biodiversity, One Health and Veterinary Medicine, College of Medical, Veterinary and Life Sciences, University of Glasgow, Glasgow, G12 8QQ Scotland; 5 Faculty of Science, University of Basel, Klingelbergstrasse 50, Basel, 4056 Switzerland; 6 Department of Bacteriology, Noguchi Memorial Institute for Medical Research, University of Ghana, P.O. Box LG 581, Accra, Ghana

**Keywords:** Wellbeing, Quality of life, Livestock farmers, Livestock diseases One health

## Abstract

**Introduction:**

Livestock production is a key livelihood source for many people in developing countries. Poor control of livestock diseases hamper livestock productivity, threatening farmers’ wellbeing and food security. This study estimates the effect of livestock mortalities attributable to disease on the wellbeing of livestock farmers.

**Methods:**

Overall, 350 ruminant livestock farmers were randomly selected from three districts located in the north, middle and southern belts of Ghana. Mixed-effect linear regression models were used to estimate the relationship between animal health and farmer wellbeing. Farmer wellbeing was assessed using the WHOQOL-BREF tool, as the mean quality-of-life in four domains (physical, psychological, social, and environmental). Animal health was assessed as annual livestock mortalities to diseases adjusted for herd size, and standardized in tropical livestock units to account for different ruminant livestock species. We adjusted for the potential confounding effect of farmers’ age, sex, educational attainment, farmland size, socio-economic status, perception of disease risk to herd, satisfaction with health, previous experience of disease outbreaks in herds, and social support availability by including these as fixed effects, and community as random effects, in a pre-specified model.

**Results:**

Our results showed that farmers had a median score of 65.5 out of 100 (IQR: 56.6 to 73.2) on the wellbeing scale. The farmers’ reported on average (median) 10% (IQR: 0 to 23) annual herd mortalities to diseases. There was a significantly negative relationship between increasing level of animal disease-induced mortality in herds and farmers’ wellbeing. Specifically, our model predicted an expected difference in farmers’ wellbeing score of 7.9 (95%CI 1.50 to 14.39) between a farmer without any herd mortalities to diseases compared to a (hypothetical) farmer with 100% of herd mortalities caused by diseases in a farming year. Thus, there is a reduction of approximately 0.8 wellbeing points of farmers, for the average of 10% disease-induced herd mortalities experienced.

**Conclusions:**

Disease-induced livestock mortalities have a significant negative effect on farmers’ wellbeing, particularly in the physical and psychological domains. This suggests that veterinary service policies addressing disease risks in livestock, could contribute to improving the wellbeing of livestock dependent populations, and public food security.

**Supplementary Information:**

The online version contains supplementary material available at 10.1186/s12889-023-16287-2.

## Introduction

Livestock production remains a key source of livelihood for many people in developing countries, particularly for rural dwellers [1]. Livestock production contributes to public food security and revenues, as well as individual-level food resources, economic prosperity, and as an asset store against uncertainty [2, 3]. In spite of its value to society, livestock production is hampered by adverse events including climate variabilities, conflicts, and animal disease outbreaks. These adversities negatively affect the productivity of the livestock sector [2].

In many sub-Saharan African countries including Ghana, transboundary animal diseases are highly prevalent due to an inadequate adoption of disease prevention and control measures, causing significant herd mortalities [4]. The lack of adequate prevention of diseases in animals predisposes humans, and the ecosystem to heightened risks of zoonotic disease, antimicrobial residue spread and related antimicrobial resistant pathogens [5, 6]. Beyond these risks to human and ecosystem health, livestock mortalities could also affect the wellbeing of livestock dependent populations. Previous research has shown a negative effect of animal disease-related mortalities on livestock farmers’ psychological wellbeing [7, 8]. Although other dimensions of the wellbeing of livestock farmers could be affected by poor animal health, there is a dearth of evidence on the extent of these effects in the literature.

Human wellbeing and productivity are closely interconnected. Research has shown a strong two-way link between productivity and wellbeing of people; better wellbeing has a strong and positive impact on productive performance in work, while the productivity gains from high performance also contribute to better wellbeing of people through higher incomes, life and job satisfaction [9]. It is essential therefore, that challenges affecting the wellbeing of working people be addressed to foster better productivity. Wellbeing could be measured either objectively or subjectively. Objective measurements of wellbeing are often implemented as aggregate population level indexes of wellbeing using different indicators such as the human development index [10], while subjective wellbeing measures involve assessment of individual’s own perception of their wellbeing [11]. The WHO Quality of Life – BREF (WHOQOL – BREF) tool is often used to assess individual’s perception of their own wellbeing including their satisfaction with the level of functioning [12].

A livestock herd’s health is measured by the herd’s productivity and ability to limit the incidence and effect of economically important diseases [13]. Although previous research has highlighted significant connections between human and animal health, the majority of existing literature has predominantly focused on areas such as the potential for zoonotic diseases, impact of antimicrobial usage on the development of pathogen resistance, and the effect of animal diseases on food security [14–19]. It is worth noting that livestock farmers share strong bonds with their animals, with livestock fulfilling additional social roles, including serving as companion animals for farmers [20, 21]. Hence, the impact of poor animal health on livestock farmers can potentially extend beyond livelihood loss, zoonotic infections, and food insecurity. Our goal in this study therefore was to evaluate the average impact on a livestock farmer’s wellbeing that could be attributed to the health and mortality of animals in the farmer’s herd.

## Materials and methods

### Description of study area

**Fig. 1:**
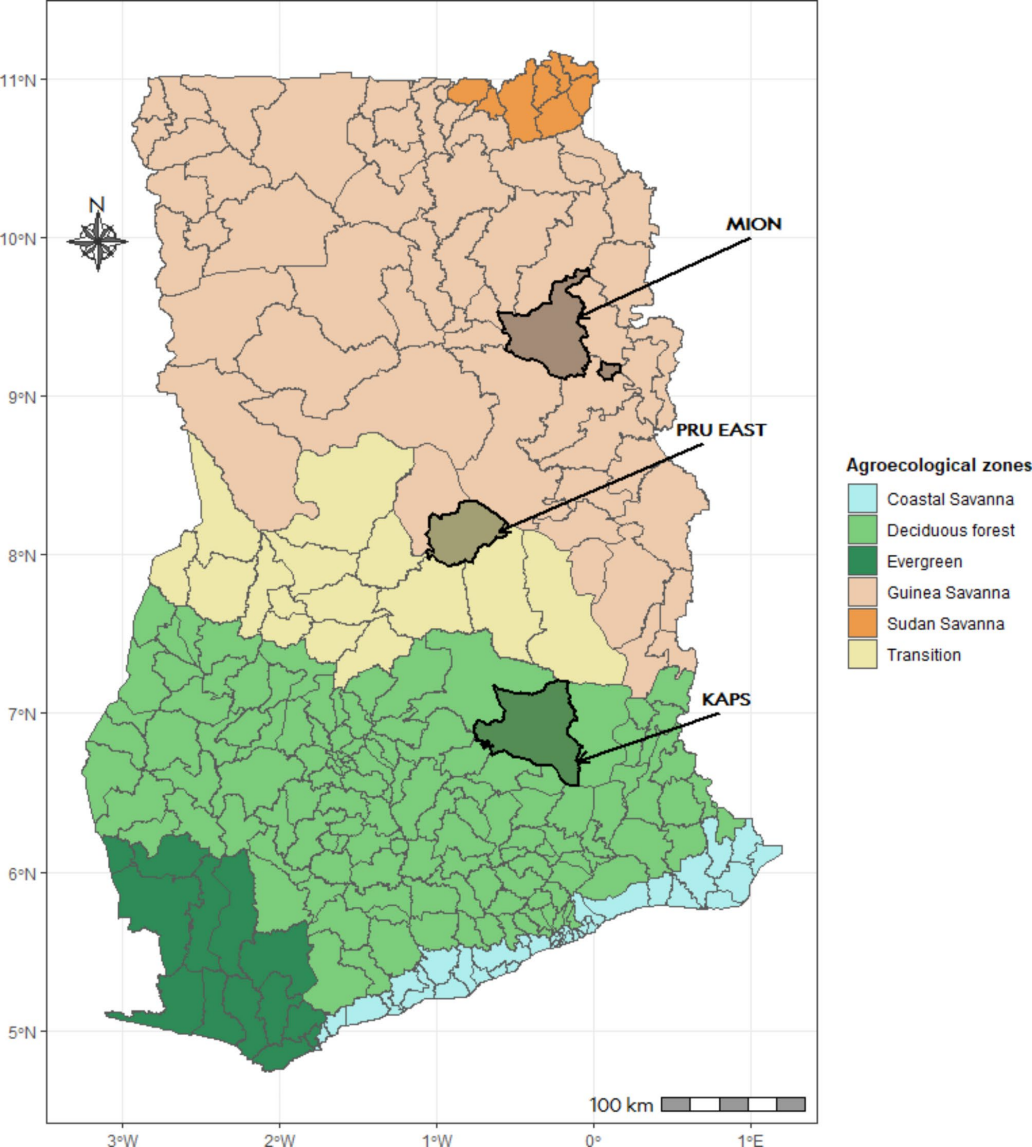
Administrative map of Ghana showing the agro-ecological zones and study districts. The figure shows the district-level administrative and ago-ecological map of Ghana. It presents the distinct locations of the study districts (shaded areas to which arrows point) within the main agro-ecological zones. MION, PRU EAST, and KAPS denote the Mion, Pru East and Kwahu Afram Plains South Districts respectively

This study was conducted in the Mion, Pru East and Kwahu Afram Plains South (KAPS) Districts, which are representative of the northern, middle and southern farming belts of Ghana. The districts lie in the Guinea Savannah, Transition and Deciduous forest Vegetation zones, which are the main livestock production zones in Ghana (Fig. 1) [22–24]. Agriculture contributed about one-fifth of the national gross domestic product of Ghana in 2019 with the livestock sector accounting for 14% of this production [25]. The selected districts are mainly rural and agrarian, with about one-third of the livestock holdings of households being ruminant species. The ruminant livestock species mainly reared by farmers include cattle, sheep, and goats. While the non-ruminant livestock species reared, include poultry, pigs, and rabbits [26].

### Study design

This study was a cross-sectional survey involving 350 ruminant livestock owners. The survey was conducted as a part of a larger project that employed a convergent parallel mixed-method design to assess the effectiveness of veterinary interventions in Ghana. The full details of the project design is provided in an earlier paper [27]. In summary, the wellbeing of the ruminant livestock farmers in the study was assessed using the WHO Quality of life – BREF tool, and herd health was assessed as the proportion of annual herd mortalities attributable to diseases. We evaluate in this paper, the sensitivity of farmer’s wellbeing to the level of disease-induced animal mortalities in the farmer’s herd, adjusting for other pre-specified covariates.

### Study population

The study population included all ruminant livestock farmers’ in the study area. We first obtained district maps and created a sampling frame of villages within the study area. Based on the population and housing census data available prior to the study (2010 Population and Housing Census), there was about 80,880, 54,694, and 47,230 tropical livestock units (TLUs) of ruminant livestock species in the KAPS, Mion and Pru East Districts respectively, with an average of about 10 holdings per household. We randomly drew from the sampling frame, 15 villages in the KAPS District, and 10 villages each in the Pru East and Mion Districts, proportional to the number of livestock farming households per district [22–24]. A household refers to a person or group of persons who normally live together and are catered for as one unit; members may or may not be related. Any member of the household who takes responsibility for the upkeep of the household’s livestock was eligible to participate in the study.

### Sample size and sampling technique

The sample size determination and sampling procedure for the survey is described in detail in an earlier paper [27]. In summary, 350 livestock farmers were recruited from 38 villages in the three study districts, proportional to the size of ruminant livestock owning households using segmentation; where selected villages are divided into smaller equal units called segments depending on size, and all eligible households recruited in one randomly selected segment [28]. In selected segments of the study villages, all households who keep ruminant livestock were eligible to be selected and the households providing consent were recruited to participate in the survey. For villages where sufficient households were not attained due to low number of livestock-owning households, the adjoining village was selected in an attempt to reach the desired sample size. Overall, the median number of farmers recruited per village was 10 farmers [interquartile range (IQR) = 7 to 11].

### Data collection and data management

The enumeration team visited the households keeping ruminant livestock to administer the questionnaires between November 2021 and January 2022. The survey questionnaire was administered to the respondents’ face-to-face using tablets with Open Data Kit (ODK) application [29]. The data collected included social support availability to farmers, farmers’ perception of disease risk to herd, farmers’ wellbeing and satisfaction with health, and other socio-demographic characteristics. The livestock farmers’ wellbeing was assessed using the WHO Quality of life – BREF (WHOQOL-BREF) tool [30]. The WHOQOL-BREF is a 26-item 5-point Likert scale, with scores ranging from 1 to 5; higher scores on the scale denote better wellbeing. Two of the items on the scale assesses the study subject’s own perception of quality of life or wellbeing and overall satisfaction with health status, and are excluded in the analysis for wellbeing. The 24 questions assess individual’s perception of their wellbeing on the physical, psychological, social, and environmental domains. Farmers’ perception of disease risk to herds was assessed on a five-item Likert scale with responses ranging from 1 to 5; higher scores indicate higher risk perception of the diseases to a herd. The social support level available to farmers was assessed on a 5-point Likert scale of the level of support, the farmers received from different facets of society including family, friends, law enforcement, credit institutions, community leaders and religious leaders, to aid them in livestock farming. We measured animal health using reported annual disease-induced mortalities of livestock relative to a herd size, and standardized in tropical livestock units [31]. The data was downloaded in Microsoft Excel format from ODK and imported into R version 4.1 [32] for analyses.

### Data analyses

We performed descriptive analyses of the survey data, comparing the distribution of responses by study district. The farmers’ herd sizes were converted to tropical livestock units (TLU) to standardize livestock holdings as follows: 1 TLU corresponds to 0.75 cattle and 0.1 small ruminants (sheep and goats) [33]. The number of animal mortalities were also converted to TLUs. The relative wealth of households was determined using an index of household’s ownership of selected assets, such as televisions, refrigerators and bicycles [34]. We determined the severity of losses suffered as the proportion of a herd lost to different factors in TLUs. The social support available to a farmer was the sum of the reported support level received from the different sources. We derived the disease risk to herd perception score as the sum of the Likert scale scores. One item score on the perception scale (Q4) is reversed to achieve a similar direction of the perception score. For the wellbeing score, firstly three negatively framed items (Q3, Q4, and Q26) were reversed to achieve a similar direction of wellbeing scores. To obtain the scores for each wellbeing domain, the mean of all items included within each wellbeing domain is calculated, and multiplied by a factor of four and then transformed to a scale from 0 to 100, according to the tool’s guidelines [30]. We derived the overall wellbeing score as the average of the four wellbeing dimension scores [35].

We performed univariable analyses, using linear regression models to compare the relationship between farmers’ wellbeing and the level of mortalities in their herds [categorized in three quantiles (tertiles): low, moderate and severe] to all causes, and specifically to diseases. We present the results using boxplots, comparing the average wellbeing scores between the levels of herd mortalities. In a pre-specified linear regression model, we evaluated the hypothesis that the level of animal disease-induced mortality in herds (herd health) is associated with farmers’ overall wellbeing, accounting for the potential confounding effects of other covariates in a linear mixed effects model. The level of disease-induced herd mortality is derived as the number of animals lost to diseases relative to each farmers’ herd size (both in TLUs). The covariates included in the model were farmers’ age, sex, educational attainment, farmland size, wealth status, perception of disease risk to herds, overall satisfaction with health, and level of social support received as fixed effects, and village-level clusters as random effects in a linear mixed effect regression model. Values of *p* < 0.05 were considered statistically significant. We performed sensitivity analysis to assess the robustness of the findings, and examined model residuals to determine if key assumptions of model fit were met.

## Results

### Socio-demographic characteristics of study respondents

**Fig. 2:**
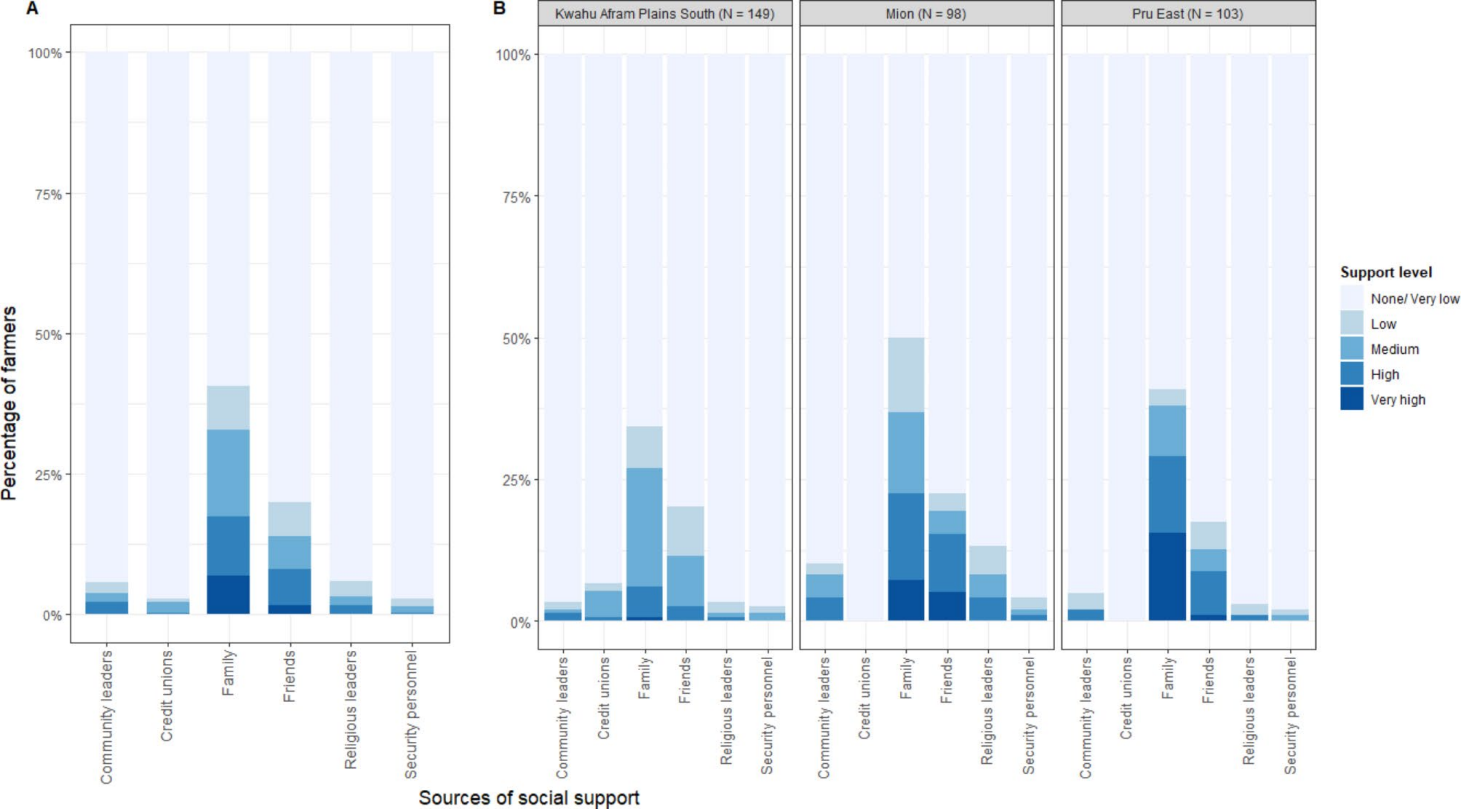
Sources and level of social support available to livestock farmers in Ghana The figure shows the distribution of support level received by farmers from different sources. **Panel A** presents the un-stratified distribution of support availability to farmers from the listed sources, while **Panel B** presents the stratified distribution of support received by study district. The height and gradient of the color shows the proportion of farmers and the level of support received from each source respectively. For the gradient of the coloration, light coloration depicts no or very low support level from a source and deep coloration depicts very high support level. The y-axis shows the proportion of the farmers receiving support from a source

Table 1 presents the socio-demographic characteristics of all study respondents (N = 350) stratified by district. The median age of the farmers completing the survey was 45 years (IQR = 35 to 54 years). The median household size was 8 persons (IQR = 6 to 11 persons), with each household keeping on average (median) 2.5 TLUs of ruminant livestock per herd (IQR = 1.3 to 7.0 TLUs). More than 95% (333/350) of the respondents own the livestock themselves. The farmers also cultivated on average 7 acres of farmland (IQR = 3 to 15 acres) in addition to rearing livestock. More than two-thirds (71%) of the respondents were male, and about half of farmers had received no formal education (51%). The wealth index analysis of households showed that in the Mion District, 67% of households were in the poorest two wealth quintiles, while the same was true only for 42% of households in KAPS and for 16% of households in the Pru East Districts. On average, farmers ranked the social support received in the study year at 6 out of 30 (IQR = 6 to 8). The social support was received mainly from family and friend sources (Fig. 2). Farmers scored on average, 19 out of 25 (IQR = 17 to 21) on the disease risk perception scale.

**Table 1:** Socio-demographic characteristics of the study respondents by study district

Characteristic	KAPS	MION	PRU EAST
	Median (IQR)	Median (IQR)	Median (IQR)
**Age (years)**	46 (36, 56)	41 (34, 51)	46 (34, 57)
**Household size (persons)**	7 (5, 10)	10 (7, 15)	8 (6, 13)
**Health satisfaction score**	75 (50, 75)	75 (50, 75)	75 (50, 75)
	**% (n/N)**	**% (n/N)**	**% (n/N)**
**Sex**			
Female	38% (57/149)	16% (16/98)	28% (29/103)
Male	62% (92/149)	84% (82/98)	72% (74/103)
**Educational attainment**			
No formal education	28% (41/149)	87% (85/98)	51% (52/103)
Up to 12 years education	48% (72/149)	6% (6/98)	28% (29/103)
Higher education	24% (36/149)	7% (7/98)	21% (22/103)
**Wealth status**			
Poorest	14% (21/149)	42% (41/98)	8% (8/103)
Below average	28% (41/149)	26% (25/98)	8% (8/103)
Average	24% (36/149)	14% (14/98)	15% (16/103)
Above average	25% (37/149)	10% (10/98)	22% (23/103)
Least poor	9% (14/149)	8% (8/98)	47% (48/103)
**Farm size (acres)**			
Small (1st tertile: 0–5 acres)	63% (94/149)	16% (16/98)	28% (29/103)
Medium (2nd tertile: 6–11 acres)	21% (31/149)	43% (42/98)	21% (22/103)
Large (3rd tertile: 12–99 acres)	16% (24/149)	41% (40/98)	51% (52/103)
**Herd size (TLU)**			
Small (1st tertile: 0.3–1.6 TLUs)	42% (62/149)	43% (42/98)	23% (24/103)
Medium (2nd tertile: 1.7–4.2 TLUs)	31% (46/149)	24% (24/98)	35% (36/103)
Large (3rd tertile: 4.3–181.9 TLUs)	27% (41/149)	33% (32/98)	42% (43/103)

For continuous variables, the median value with corresponding lower and upper quartile values (IQR) are presented in parentheses. Percentages (%) are the proportions of ruminant livestock farmers within each characteristic explored per study district sub-sample (N). Numbers (n) of farmers, falling into each sub-category of characteristics within the study districts; Kwahu Afram Plains South (KAPS), Mion and Pru East Districts

### Effect of livestock mortality on livestock farmers’ wellbeing

**Fig. 3:**
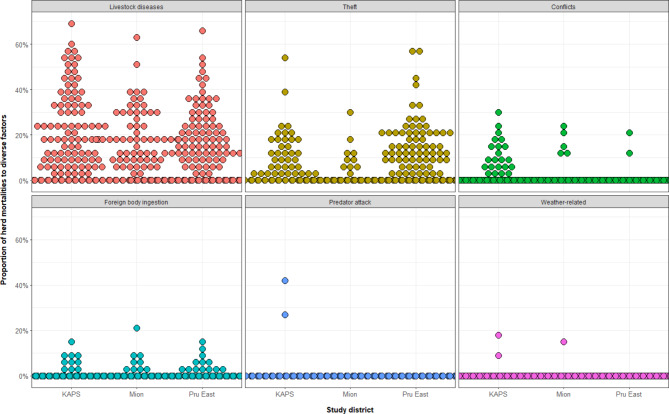
Factors causing animal mortality in ruminant livestock herds in Ghana Presents the distribution of the proportion of farmers’ herds lost to different factors. The y-axis shows the proportion of herd mortalities for each specified factor depicted by different colors for a livestock farmer and stratified by study district. The position of each dot on the y-axis denotes each individual farmer’s level of reported losses to a factor

The farmers reported a median of 0.5 TLUs (IQR = 0.1 to 1.4 TLUs) of ruminant livestock mortalities per herd in the study year (2021), corresponding to an average (median) of 19% mortality per herd (IQR = 6 to 37%). Livestock diseases accounted for the majority of reported herd mortalities. The farmers reported a median disease-induced mortalities of 10% of the herds (IQR = 0 to 23%) (Fig. 3). About 45% (159/350) of farmers had past history of disease outbreaks in their herds, while 47% (164/350) of them reported a disease outbreak in the study year (2021).

Table 2 presents the farmers’ scores on the physical, psychological, social and environmental domains of wellbeing, as well as a pooled overall wellbeing score. The farmers scored on average (median) 71.4 out of 100 (IQR = 57.1 to 85.7) on the physical, 70.8 out of 100 (IQR = 58.3 to 79.2) on the psychological, 66.7 out of 100 (IQR = 50.0 to 75.0) on the social and 56.3 out of 100 (IQR = 43.8 to 65.6) on the environmental domains of wellbeing. The median overall wellbeing score was 65.5 out of 100 (IQR = 56.6 to 73.2). The farmers ranked their overall satisfaction with health at 75 out of 100 on average (IQR = 50 to 75).

**Table 2:** Summary of overall wellbeing and wellbeing domain scores by study district

Domain	Number of items	KAPS	MION	PRU EAST
		Median (IQR)	Median (IQR)	Median (IQR)
Overall wellbeing	24	65.1 (55.8, 72.5)	67.0 (60.3, 76.3)	64.8 (55.4, 72.2)
Physical	7	71.4 (53.6, 82.1)	82.1 (67.9, 89.3)	67.9 (53.6, 78.6)
Psychological	6	66.7 (58.3, 79.2)	75.0 (62.5, 83.3)	66.7 (58.3, 75.0)
Social	3	66.7 (58.3, 75.0)	66.7 (58.3, 83.3)	58.3 (50.0, 75.0)
Environment	8	53.1 (43.8, 62.5)	50.0 (40.6, 62.5)	59.4 (50.0, 68.8)

Wellbeing domains include physical, psychological, social and environmental quality of life of farmers assessed using the WHO Quality of life – BREF tool; a 24-item 5-point Likert scale. Overall wellbeing is the average of scores in all the domains of wellbeing. Median wellbeing scores with corresponding interquartile ranges (IQR) stratified by study district are presented

**Fig. 4:**
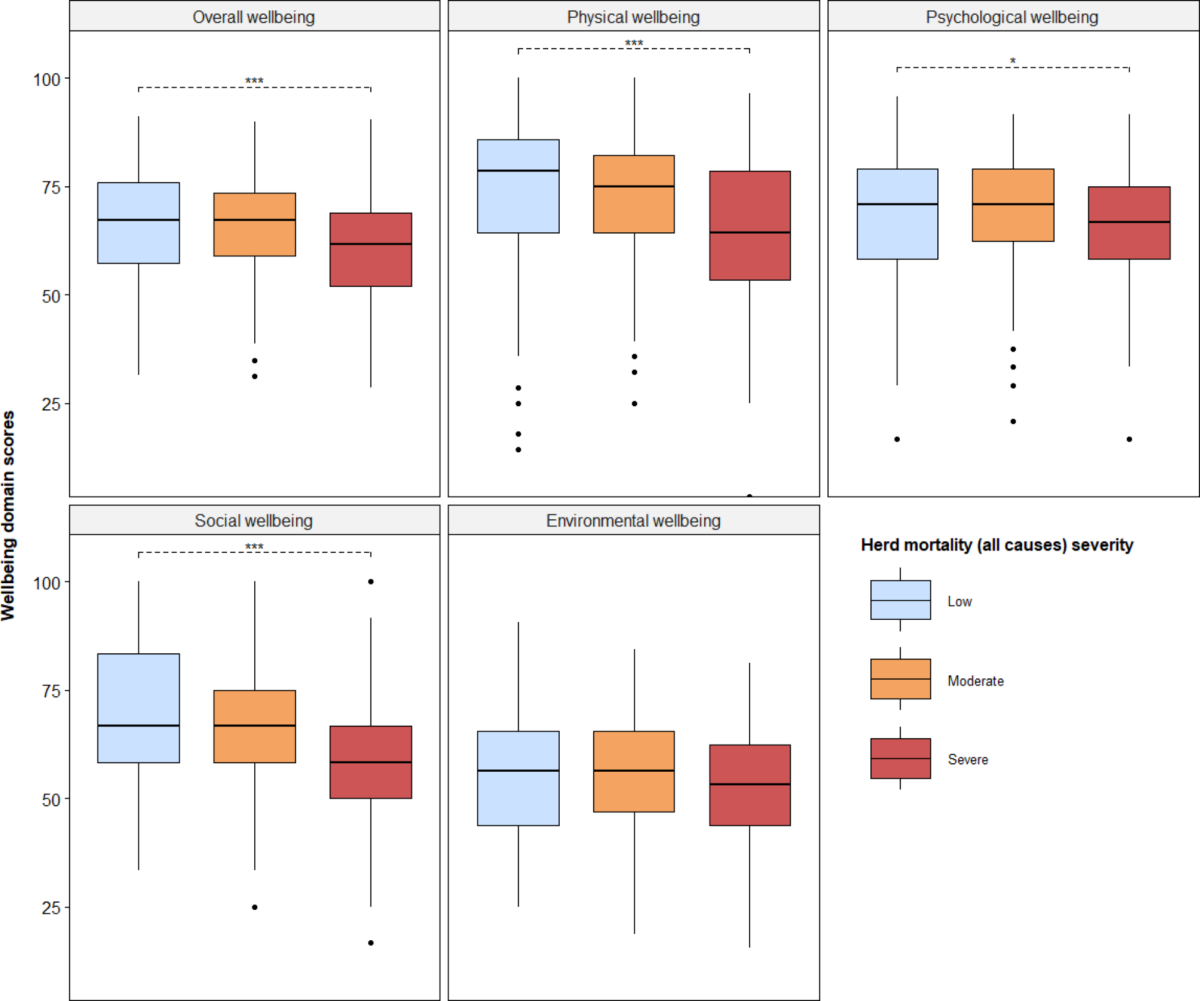
Relationship between herd mortality to all causes and farmers’ wellbeing Shows the relationship between the level of herd mortality to all causes and farmers’ wellbeing in all domains. The overall wellbeing is the average of wellbeing scores in the physical, psychological, social and environmental domains. The level of herd mortalities are reported animal deaths on farms due to all causes relative to a farmer’s herd size in the study year. The level of herd mortality is categorized into tertiles (three quantiles) of severity: low (less than 1% of herd mortality), moderate (1 to 30% of herd mortality) and severe (more than 31% of herd mortality). The box plots show the average wellbeing scores with corresponding interquartile ranges for farmers within each level of herd mortality, with the levels of herd mortalities distinguished by colors. The dashed lines show significant results of hypothesis testing of the relationship between farmers’ wellbeing and higher levels of herd mortalities compared to low loss levels using a linear regression model. *, ***, denote 5%, and 0.1% significance levels respectively

We assessed the relationship between the level of mortality in herds and overall farmer wellbeing. The levels of herd mortality to all causes and specifically to diseases, was categorized into tertiles (three quantiles); low, moderate and severe, based on the distribution of proportions of herd mortalities. Figure 4 presents the relationship between farmers’ wellbeing in all domains and the three levels of herd mortalities (low, moderate and severe) to all causes. Farmers with severe herd mortalities (more than 31% of herd mortality) had significantly lower levels of overall (mean score of 60.5 versus 66.5, *p* < 0.001), physical (64.1 vs. 73.4, *p* < 0.001), psychological (64.6 vs. 69.2, *p* = 0.04), and social (60.2 vs. 68.2, *p* < 0.001) wellbeing, compared to farmers with low level of loss (less than 1% of herd mortality).

**Fig. 5:**
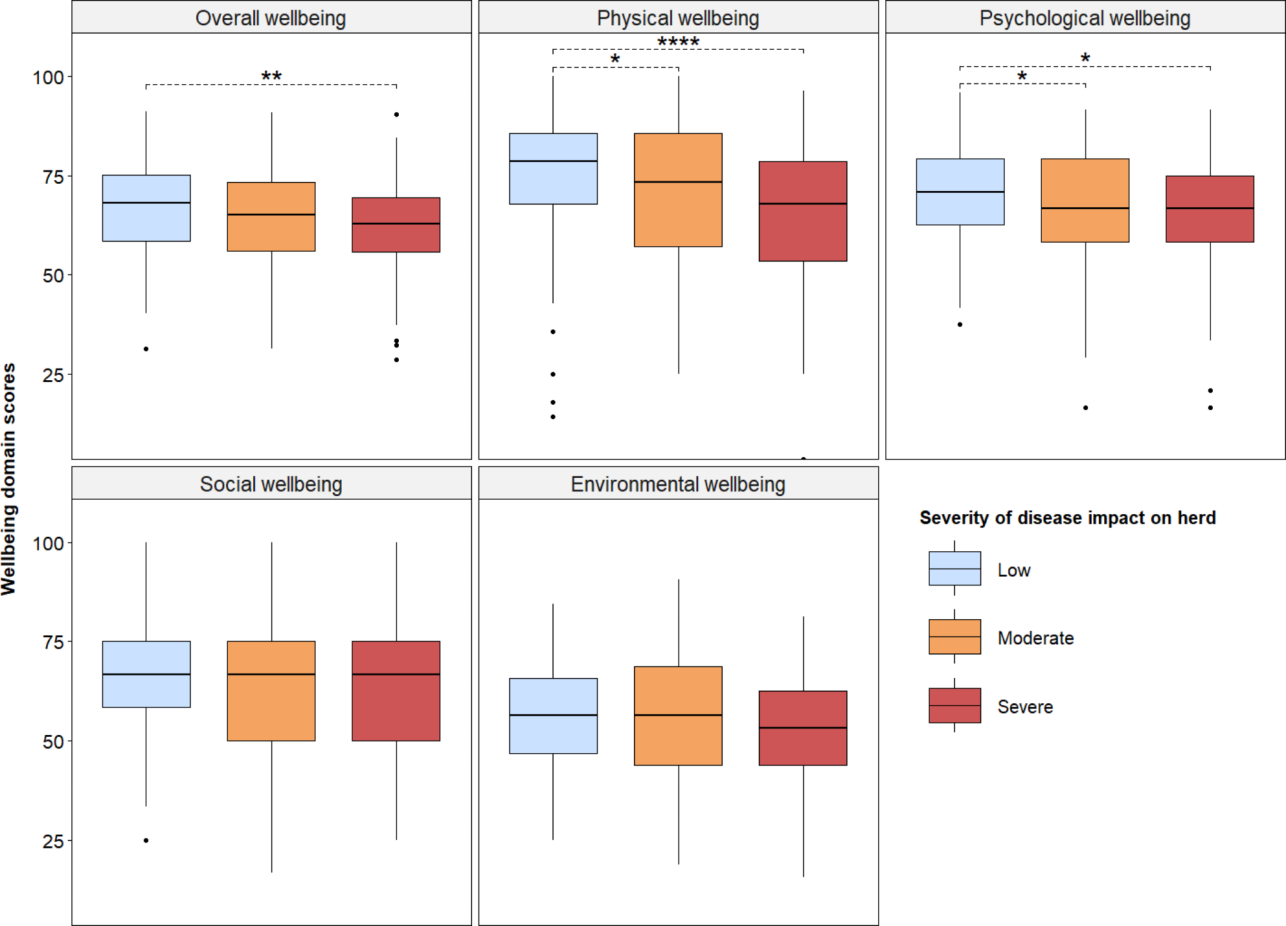
Relationship between herd mortality to diseases and farmers’ wellbeing shows the relationship between the level of herd mortality specifically to only diseases and farmers’ wellbeing in all domains. The overall wellbeing is the average score of wellbeing scores in the physical, psychological, social and environmental domains. The level of herd mortalities are reported disease-induced animal deaths on farms relative to a farmer’s herd size in the study year. The level of herd mortality is categorized into tertiles (three quantiles) of severity: low (less than 1% of herd mortality), moderate (1 to 18% of herd mortality) and severe (more than 18% of herd mortality). The box plots show the average wellbeing scores with corresponding interquartile ranges for farmers within each level of herd mortality, with the levels of disease-induced herd mortalities distinguished by colors. The dashed lines show significant results of hypothesis testing of the relationship between farmers’ wellbeing and higher levels of herd mortalities to diseases compared to low loss levels using a linear regression model. *, **, ***, denote 5%, 1%, and 0.1% significance levels respectively

The relationship between levels of herd mortalities specific to diseases and farmers’ wellbeing is presented in **Fig. **5. The level of disease-induced herd mortalities was significantly associated with farmers’ overall, physical, and psychological wellbeing. The farmers with severe herd losses (more than 18% of herd mortality to diseases), had significantly lower overall (mean score of 61.7 versus 66.9, *p* = 0.002), physical (65.1 vs. 74.6, *p* < 0.001), and psychological (65.7 vs. 70.6, *p* = 02) wellbeing scores, compared to the farmers with low level of losses (less than 1% of herd mortality to diseases). While farmers with moderate herd losses (between 1% and 18% of herd mortality) also had significantly lower physical (69.4 vs. 74.6, *p* = 0.02) and psychological (66.7 vs. 70.6, *p* = 0.04) wellbeing scores compared to the farmers with low level of loss.

Table 3 presents the results of the linear mixed effect regression model, with fixed effects for disease-related herd mortalities relative to the herd sizes (all in TLUs), farmers’ age, sex, educational attainment, farmland size, wealth index, social support level received, overall satisfaction with health, and perception of disease risk to herd and village-level clusters as random effects.

**Table 3:** Mixed effects model predicting the effect of level of herd mortalities to diseases on farmer wellbeing scores adjusting for other covariates

Parameter	Estimate	95% CI	*p*-value
Fixed effects			
Proportion of herd mortality *	-7.94	-14.39 – -1.50	0.02
**Satisfaction with health**	0.27	0.23–0.32	< 0.001
**Social support received**	0.84	0.47–1.22	< 0.001
**Perception of disease risk to herd**	0.41	0.01–0.80	0.04
**Age (years)**	-0.10	-0.17 – -0.02	0.01
**Farm size (acres)**	0.06	-0.02–0.14	0.17
**Sex** [ref = female]			
Male	1.50	-0.72–3.71	0.19
**Education level** [ref = no formal education]			
Up to 12 years	0.01	-2.36–2.37	0.99
Higher education	2.27	-0.53–5.07	0.11
**Wealth index** [ref = poorest]			
Below average	0.08	-2.93–3.10	0.96
Average	2.80	-0.36–5.95	0.08
Above average	2.92	-0.38–6.23	0.08
Least poor	2.92	-0.53–6.38	0.09
**History of disease outbreak** [ref = No]			
Yes	-0.01	-2.21–2.20	0.99
**Random effects**			
**Within cluster standard deviation**	8.78	8.02–9.56	…
**Between cluster standard deviation**	2.08	0.00–3.44	…
*Marginal R* ^*2*^ */ Conditional R* ^*2*^	0.47 / 0.50		

* Proportion of herd mortality refers to livestock mortalities to diseases relative to herd size standardized in tropical livestock units. Estimates are the mean changes in overall wellbeing scores of ruminant livestock farmers attributable to changes in parameters, with their corresponding 95% confidence intervals (95% CI) and p-values. Overall wellbeing is the average of scores in all the wellbeing domains including physical, psychological, social and environmental wellbeing assessed using the WHO Quality of life – BREF tool. “ref” denotes the reference level for categorical variables in the model. Marginal and conditional *R*^*2*^ are the model variance explained by the fixed effect, and both fixed and random effects respectively

**Fig. 6:**
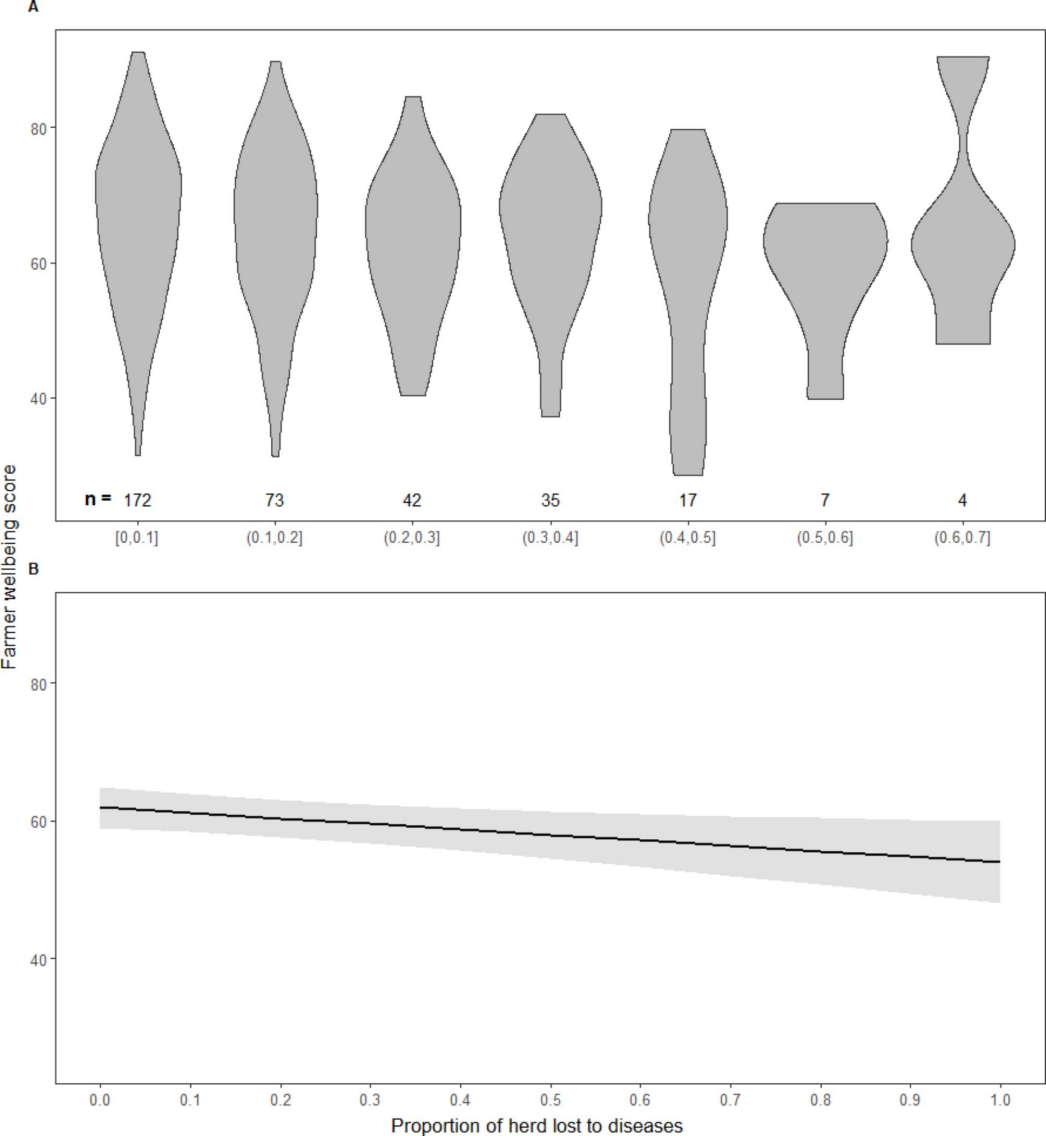
Effect of herd mortalities to diseases on farmers’ wellbeing Shows the actual and predicted relationship between the severity of disease-induced animal mortalities and farmers’ overall wellbeing. The overall wellbeing is the average score of wellbeing scores in the physical, psychological, social, and environmental domains. **Panel A** shows the relationship between 10% increments in relative herd mortalities to diseases and farmers overall wellbeing without accounting for the potential confounding effect of other covariates. **Panel B** shows the estimated marginal effect at different levels of disease-induced livestock mortalities, conditional on the other co-variates in the pre-specified linear mixed effect linear regression model. The slope of the marginal effect line with confidence intervals around the point estimates shows the extent and direction of the relationship between the levels of disease-induced herd mortalities and livestock farmers’ overall wellbeing

There was a significantly negative relationship between increasing levels of disease-induced herd mortalities and farmers’ overall wellbeing. Specifically, our model predicted an expected difference in farmers’ wellbeing score of 7.9 (95%CI 1.50 to 14.39) between a farmer without any animal mortalities compared to a hypothetical farmer with 100% of animal mortalities to diseases. Thus, there is a reduction of approximately 0.8 wellbeing points of farmers, for the average of 10% disease-induced herd mortalities experienced (Fig. 6). A likelihood-ratio test showed that the model including disease-induced herd mortalities provided a better fit for the data than a model without it, 𝒳^2^[1] = 6.13, *p* = 0.01. Excluding livestock farmers who did not own animals in their herds did not change the results and conclusions (**Additional file 1**). In addition, including the other causes of animal mortalities relative to the herd size did not change significantly the effect size (**Additional file 2**).

## Discussion

In this study, we aimed to estimate the average effect on the wellbeing of a livestock farmer that can be attributed to disease-induced mortalities in the farmer’s herd. To achieve this goal, we adopted a cross-sectional survey design, in which we measured farmers’ wellbeing and annual herd mortalities and evaluated this association, accounting for specified covariates, using linear mixed effect models. Our results suggest that the level of animal disease-induced herd mortalities have a large and negative effect on farmers’ wellbeing significantly different from zero, particularly in the physical and psychological domains of wellbeing. The effect size did not change significantly after the inclusion of other causes of livestock mortality including theft, conflict, accidents and weather-related herd mortalities and control variables in the model.

These results underscore the need to consider the interdependencies between human, animal and ecosystem health, beyond zoonosis spread in health research. There exists substantial evidence supporting the impact on global health security, of pathogen spread between the animal, human, and environmental interfaces, in the absence of adequate control measures [6]. These health impact evaluations usually have a biomedical physical health focus. Thus, the observed impact could be even larger when the multidimensionality of health is fully considered. We have demonstrated in this study that the impact of poor animal health on farmers’ overall wellbeing is large and significant. Few studies have highlighted the strong link between poor animal health and the psychological wellbeing of livestock dependent populations [7, 8, 36, 37].

The effect of the severity of herd mortalities to diseases was more pronounced on the physical and psychological domains of health compared to the other wellbeing domains (i.e. social and environmental wellbeing). This finding is intuitive given the extensive nature of farming in the study area [26] and the relative emotional and security attachment between farmers and their livestock [7, 21, 38]. Other sources of herd mortality including livestock theft, conflict, and weather-related losses would affect more the social and environmental domains of wellbeing, compared to disease-induced losses as shown in our results. The extent of these associations could be assessed in future studies. In-depth studies from an ecosystem perspective, of the relationship between ecosystem challenges, and human and animal wellbeing, are needed.

Disease-induced livestock mortalities remain a significant barrier to the productivity and trade in the livestock sector in many African countries including Ghana [4]. Similar to our results, previous research in other countries identified animal diseases as a significant source of livestock herd mortalities for households [39–42]. Based on this impact of diseases on herds, studies have emphasized the effectiveness and profitability of applying preventive measures particularly vaccination to sustainably address disease-induced livestock mortalities [43–46]. Our findings in the earlier studies of the larger project showed that the main diseases causing livestock mortalities are Contagious Bovine Pleuropneumonia and Food and Mouth Disease in cattle, and Peste des Petits Ruminants in small ruminants (sheep and goats) [27]. Vaccination utilization by farmers to protect herds against these diseases was also very low [47] although observed as the key intervention that reduces the mortalities [43]. There is thus the need for transdisciplinary strategies that improve high quality vaccine adoption, given the availability of effective vaccines to control these diseases [48]. The evidence from our work suggests that, addressing animal health challenges through veterinary service policies could contribute to improving the wellbeing of livestock dependent populations. However, it should be noted that disease control policies should be adequate to the farming systems. For example the mass culling of livestock during the Foot and Mouth Disease epidemic in the United Kingdom led to larger mental health and suicide problems [49]. In this particular instance, a ring vaccination and quarantine policy might have been more appropriate.

Our study had some limitations. The nature of the design does not enable us to determine the temporal relationship between poor animal health (disease-induced mortalities) and farmer wellbeing. Furthermore, in our attempt to measure reliably the impact of diseases on farmers’ herds, we relied on only disease-induced herd mortalities. Thus, the impact of diseases resulting in only morbidity without the death of the infected animals was not accounted for in our measurements. We argue however that, the observed impact on farmers’ wellbeing is likely to be larger, if disease-induced morbidities should be considered. Future studies implementing interventions to reduce disease incidence using randomized controlled trials could evaluate the extent of this relationship more definitively, as well as assess the pathways of the impact. Our study focused on ruminant livestock farmers, however, based on our engagements with the farmers in our study who also own other species such as poultry and pigs, we understand that they experience similar challenges with diseases among these other species. Thus, future studies could further explore this missing perspective in our study. Additionally, despite efforts to obtain a representative sample of the different agro-ecological zones in Ghana, our study did not account for the two other minority agro-ecological zones namely the Evergreen and Coastal Savannah zones. Even though these zones are not typical areas for livestock production in Ghana, their inclusion would have improved the representativeness of our findings with diversification and the crop production as adaptations options. In spite of this missing perspective, we do not expect the parameters evaluated to be markedly different in these agro-ecological zones. Our study thus, has provided valuable information on the relationship between poor animal health and the wellbeing of livestock dependent populations, making a strong case for improvements in performance of veterinary services, for better animal health.

## Conclusion

Our study has shown that diseases are the main cause of animal mortalities for ruminant livestock farmers in Ghana. The poor health of the livestock herds has a significant influence on the wellbeing of the livestock farmers. Given that, the main diseases accounting for these mortalities have effective vaccines for their control, and vaccination utilization is low among the farmers, our findings suggest that improvements in veterinary policies and service delivery, which address disease risks in livestock, would contribute to better wellbeing of livestock dependent populations. This study exemplifies the benefits of integrated human and animal health studies through a One Health approach, which cannot be achieved if human and animal health are studied in separation.

## Electronic supplementary material

Below is the link to the electronic supplementary material.

**Table S1**: Mixed effects model predicting the effect of level of herd mortalities to all causes on farmer wellbeing adjusting for other covariates. **S1 Figure**: Effect of the level of herd mortalities to diseases on farmers’ wellbeing (N = 333) The figure shows the actual and predicted relationship between the level of animal mortalities to diseases and farmers’ overall wellbeing. The overall wellbeing is the average score of wellbeing scores in physical, psychological, social, and environmental domains. Panel A shows the relationship between 10 percentage increments in relative herd mortalities to diseases and farmers overall wellbeing without accounting for the potential confounding effect of other covariates. Panel B presents the estimated marginal effect at different levels of disease-induced livestock mortalities, conditional on the other co-variates in the pre-specified linear mixed effect model. The slope of the marginal effect line with confidence intervals around the point estimates shows the extent and direction of the relationship between the levels of disease-induced herd mortalities and livestock farmers’ overall wellbeing

**Table S2**: Mixed effects model predicting the effect of level of herd mortalities to all causes on farmer wellbeing adjusting for other covariates. **S2 Figure**: Effect of the level of herd mortalities suffered on farmers’ wellbeing The figure shows the actual and predicted relationship between the level of animal mortalities to all causes and farmers’ overall wellbeing. The overall wellbeing is the average score of wellbeing scores in physical, psychological, social, and environmental domains. Panel A shows the relationship between 10 percentage increments in relative herd mortalities to all causes and farmers overall wellbeing without accounting for the potential confounding effect of other covariates. Panel B presents the estimated marginal effect at different levels of livestock mortalities experienced, conditional on the other co-variates in the pre-specified linear mixed effect model. The slope of the marginal effect line with confidence intervals around the point estimates shows the extent and direction of the relationship between the levels of herd mortalities to all causes and livestock farmers’ overall wellbeing

## Data Availability

All data generated or analyzed during this study are included in this published article [and its supplementary information files].
