# Supplementary material for: Relationship between animal health and livestock farmers’ wellbeing in Ghana: beyond zoonoses
Source: BMC Public Health. 2023 Jul 14;23:1353. doi: 10.1186/s12889-023-16287-2 (PMC10347735; doi:10.1186/s12889-023-16287-2)
Supplement: Supplementary file 1 — Table S1: Mixed effects model predicting the effect of level of herd mortalities to all causes on farmer wellbeing adjusting for other covariates. S1 Figure: Effect of the level of herd mortalities to diseases on farmers’ wellbeing (N = 333) The figure shows the actual and predicted relationship between the level of animal mortalities to diseases and farmers’ overall wellbeing. The overall wellbeing is the average score of wellbeing scores in physical, psychological, social, and environmental domains. Panel A shows the relationship between 10 percentage increments in relative herd mortalities to diseases and farmers overall wellbeing without accounting for the potential confounding effect of other covariates. Panel B presents the estimated marginal effect at different levels of disease-induced livestock mortalities, conditional on the other co-variates in the pre-specified linear mixed effect model. The slope of the marginal effect line with confidence intervals around the point estimates shows the extent and direction of the relationship between the levels of disease-induced herd mortalities and livestock farmers’ overall wellbeing [file 12889_2023_16287_MOESM1_ESM.docx]

**Table S1**: Mixed effects model predicting the effect of level of herd mortalities to all causes on farmer wellbeing adjusting for other covariates

| **Parameter** | **Estimate** | **95% CI** | ***p*-value** |
| --- | --- | --- | --- |
| **Fixed effects** |  |  |  |
| **Proportion of herd mortality *** | -9.02 | -15.51 – -2.53 | 0.01 |
| **Satisfaction with health** | 0.26 | 0.22 – 0.31 | <0.001 |
| **Social support received** | 0.93 | 0.55 – 1.31 | <0.001 |
| **Perception of disease risk to herd** | 0.38 | -0.03 – 0.79 | 0.07 |
| **Age (years)** | -0.08 | -0.16 – -0.01 | 0.03 |
| **Farm size (acres)** | 0.05 | -0.03 – 0.13 | 0.22 |
| **Sex** [ref = female] |  |  |  |
| Male | 1.33 | -0.91 – 3.57 | 0.24 |
| **Education level** [ref = no formal education] |  |  |  |
| Up to 12 years | 0.09 | -2.34 – 2.52 | 0.94 |
| Higher education | 2.32 | -0.50 – 5.13 | 0.11 |
| **Wealth index** [ref = poorest] |  |  |  |
| Below average | 0.32 | -2.75 – 3.38 | 0.84 |
| Average | 3.00 | -0.14 – 6.13 | 0.06 |
| Above average | 3.35 | 0.06 – 6.65 | 0.04 |
| Least poor | 2.79 | -0.67 – 6.24 | 0.11 |
| **History of disease outbreak**  [ref = No] |  |  |  |
| Yes | 0.11 | -2.14 – 2.36 | 0.92 |
| **Random effects** |  |  |  |
| **Within cluster standard deviation** | 8.68 | 7.98 – 9.41 | … |
| **Between cluster standard deviation** | 2.08 | 0.00 – 3.58 | … |
| *Marginal R^2^ / Conditional R^2^* | 0.46 / 0.49 | |  |

* Proportion of herd mortality refers to total livestock mortalities relative to herd size, standardized in tropical livestock units. Estimates are the changes in overall wellbeing scores of ruminant livestock farmers attributable to changes in parameters, with their corresponding 95% confidence intervals (95% CI) and p-values. Overall wellbeing is the average of scores in all the wellbeing dimensions including physical, psychological, social and environmental wellbeing assessed using the WHO Quality of life – BREF tool. The farmers included in the analysis are only those who own the animals in the herds (N = 333). “ref” denotes the reference level for categorical variables in the model. Marginal and conditional *R^2^* are the model variance explained by the fixed effect, and both fixed and random effects respectively.


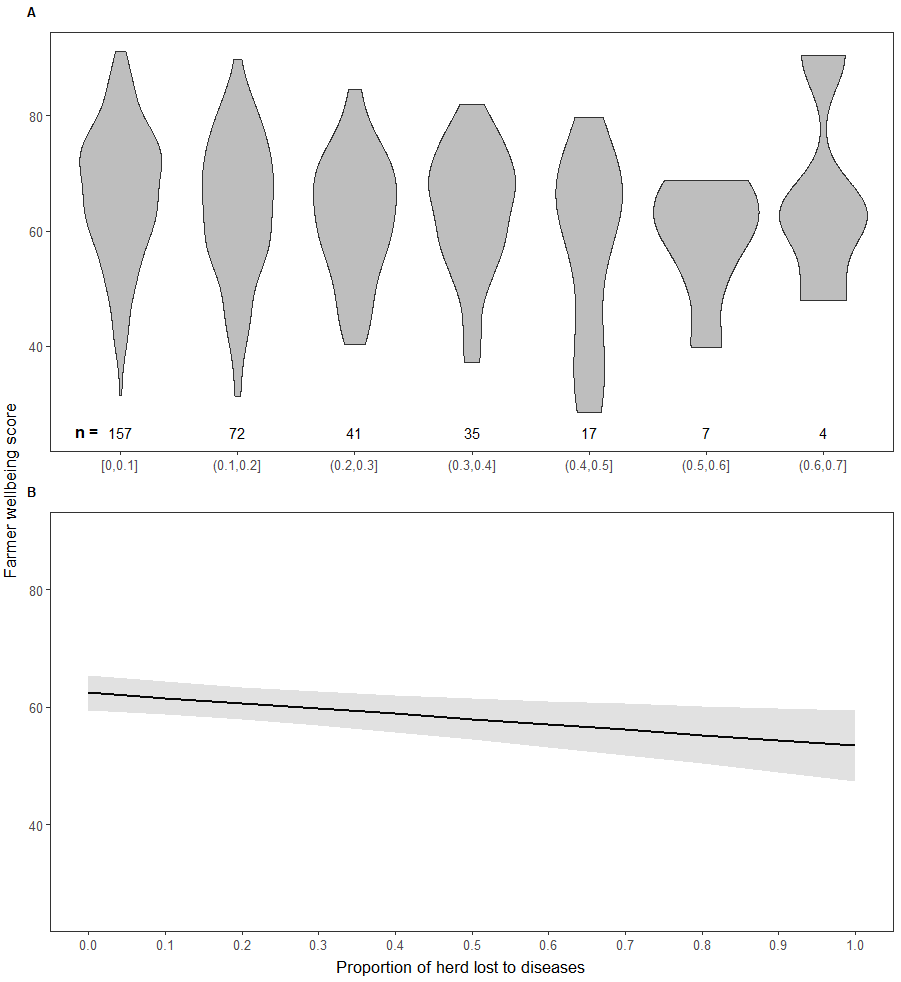


**S1 Figure:** Effect of the level of herd mortalities to diseases on farmers’ wellbeing (N = 333)

The figure shows the actual and predicted relationship between the level of animal mortalities to diseases and farmers’ overall wellbeing. The overall wellbeing is the average score of wellbeing scores in physical, psychological, social, and environmental domains. **Panel A** shows the relationship between 10 percentage increments in relative herd mortalities to diseases and farmers overall wellbeing without accounting for the potential confounding effect of other covariates. **Panel B** presents the estimated marginal effect at different levels of disease-induced livestock mortalities, conditional on the other co-variates in the pre-specified linear mixed effect model. The slope of the marginal effect line with confidence intervals around the point estimates shows the extent and direction of the relationship between the levels of disease-induced herd mortalities and livestock farmers’ overall wellbeing.
